# Anaphylaxis to Iodinated Contrast Media: Clinical Characteristics Related with Development of Anaphylactic Shock

**DOI:** 10.1371/journal.pone.0100154

**Published:** 2014-06-16

**Authors:** Min-Hye Kim, Suh-Young Lee, Seung-Eun Lee, Min-Suk Yang, Jae-Woo Jung, Chang Min Park, Whal Lee, Sang-Heon Cho, Hye-Ryun Kang

**Affiliations:** 1 Department of Internal Medicine, Division of Allergy and Clinical Immunology, Seoul National University Hospital, Seoul, Republic of Korea; 2 Institute of Allergy and Clinical Immunology, Seoul National University Medical Research Center, Seoul, Republic of Korea; 3 Seoul National University Hospital Regional Pharmacovigilance Center, Seoul, Republic of Korea; 4 Department of Internal Medicine, Ewha Womans University School of Medicine, Seoul, Republic of Korea; 5 Department of Internal Medicine, SMG-SNU Boramae Medical Center, Seoul, Republic of Korea; 6 Department of Internal Medicine, Chung-Ang University College of Medicine, Seoul, Republic of Korea; 7 Department of Radiology and Institute of Radiation Medicine, Seoul National University College of Medicine, Seoul, Republic of Korea; Centre de Recherche Public de la Santé (CRP-Santé), Luxembourg

## Abstract

**Objective:**

Anaphylaxis is the most severe form of radiocontrast media (RCM) induced hypersensitivity and can be life-threatening if profound hypotension is combined. With increased use of iodine based RCM, related hypersensitivity is rapidly growing. However, the clinical characteristics and risk factors of RCM induced anaphylaxis accompanied by hypotension (anaphylactic shock) are not clearly defined. This study was performed to investigate the risk factors of RCM induced anaphylactic shock and the clinical value of RCM skin testing to identify causative agents in affected patients.

**Methods:**

We analyzed the data of RCM induced anaphylaxis monitored by an inhospital pharmacovigilance center at a tertiary teaching hospital from January 2005 to December 2012 and compared the clinical features and skin test results according to the accompanying hypotension.

**Results:**

Among total of 104 cases of RCM induced anaphylaxis, 34.6% of patients, developed anaphylaxis on their first exposure to RCM. Anaphylactic patients presenting with shock were older (57.4 vs. 50.1 years, *p* = 0.026) and had a history of more frequently exposure to RCM (5.1±7.8 vs. 1.9±3.3, *p* = 0.004) compared to those without hypotension. Among RCMs, hypotension was more frequent in anaphylaxis related to iopromide compared to other agents (85.0% vs. 61.4%, *p* = 0.011). Skin tests were performed in 51 patients after development of RCM induced anaphylaxis. Overall skin test positivity to RCM was 64.7% and 81.8% in patients with anaphylactic shock.

**Conclusion:**

RCM induced anaphylactic shock is related to multiple exposures to RCM and most patients showed skin test positivity to RCM.

## Introduction

Anaphylaxis is a rapid-onset severe hypersensitivity reaction that can be fatal. Although death from anaphylaxis is not common and most episodes of anaphylaxis can be reversed by a single dose of epinephrine, severe anaphylaxis accompanied with cardiovascular collapse can be resistant to treatment and result in death.

As the use of computed tomography (CT) is rapidly growing, iodine based radiocontrast media (RCM) is administered about 75,000,000 times per year worldwide [1]. As low osmolality non-ionic contrast agents replaced high-osmolality ionic ones, the incidence of immediate RCM hypersensitivity diminished remarkably from 3.8–12.7% to 0.7–3.1% [2]–[4]. Similarly, the incidence of severe immediate RCM hypersensitivity also decreased from 0.1–0.4% to 0.01–0.04%. However, anaphylactic deaths still occur in 1–3 per 100,000–1,000,000 administrations regardless of ionicity [5], [6]. Presently, the clinical characteristics and risk factors for the development of anaphylactic shock are not clearly defined.

The principle of post-anaphylaxis management is to avoid the causative agents. Although other imaging can be used as an alternative test in RCM hypersensitivity patients, CT imaging has its own advantage and unavoidable in some clinical situations. Although antihistamines and systemic steroids can be used as preventive measures, they cannot ensure complete prevention of RCM induced anaphylaxis [7], [8]. Currently, there are no established guidelines on premedication for RCM induced anaphylaxis [9]. Therefore, information on a causal agent and safer substitutes will be very useful for patients who need contrast enhanced CT scan despite previous history of RCM induced anaphylaxis. Until recently, diagnostic value of RCM skin test has been underestimated and there are only a limited number of studies which evaluated the sensitivity of RCM skin testing to various RCM [10]–[12].

This study was performed to investigate the risk factors for the development of hypotension and the clinical value of RCM skin testing to identify causative agents in RCM induced anaphylaxis.

## Methods

### 1. Study Subjects

This study protocol was approved by the institutional review board (IRB) of Seoul National University Hospital. Informed consents of patients were exempted from IRB because this study only used retrospective chart review data and all personal data was eliminated and coded as arbitrary number which were not personally-identifiable. Research data was accessed only by researchers using password.

We extracted all the cases of RCM induced hypersensitivity based on ATC code of causative agents (V08A: X-ray contrast media, iodinated, V08B: X-ray contrast media, non-iodinated)’ and WHOART (ARRN: 0712 allergic reaction, 0713 anaphylactic shock, 0714 anaphylactoid reaction, 2237 anaphylactic reaction, 2268 documented hypersensitivity to administered drug) from our inhospital pharmacovigilance database collected from January 2005 to December 2012 at Seoul National University Hospital, in Seoul, Korea. Demographic and clinical data of affected patients such as age, sex, number of contrast exposures, laboratory test results, and underlying diseases based on ICD-10 were collected from electronic medical records. This study dealt only with CT procedures, not with other procedures such as cardiac catheterization or coronary angiography.

All the medical records were thoroughly re-evaluated by two allergy specialists to assess clinical features of anaphylaxis and the presence of previous contrast hypersensitivity reactions. Anaphylaxis was diagnosed if cases satisfied the criteria of anaphylaxis suggested by the National Institute of Allergy and Infectious Disease and Food Allergy and the Anaphylaxis Network [13]. Hypotension was considered as a systolic blood pressure less than 90 mmHg or greater than 30% decrease from an individual’s baseline. [13] Hypotension unrelated with underlying diseases or other drugs was considered a manifestation of anaphylaxis.

After completing review, patients with anaphylaxis were classified into two groups depending on combined hypotension. We analyzed the data to identify risk factors for the development of anaphylactic shock by comparing anaphylactic patients combined with and without hypotension.

### 2. Skin Tests with Iodinated Contrast Agents

Skin tests were carried out after experiencing RCM induced anaphylaxis for those patients who agreed to undergo skin testing. Skin prick and intradermal tests were performed on the volar part of the forearm with 6 different RCM used in our hospital - iopromide (Ultravist®, Bayer Healthcare, Brussels, Belgium), iopamidol (Pamiray®, Dongkook Pharm. Co., Ltd, Korea), iomeprol (Iomeron®, Bracco, Milan, Italy), iohexol (Omnipaque®, Armersham Health, Princeton, NJ), iodixanol (Visipaque®, Armersham Health, Princeton, NJ), and iobitridol (Xenetics®, Guerbet, Gorinchem, Netherlands). Undiluted solution and 1∶10 diluted solution were used for the skin prick test and intradermal test, respectively, as used in previous studies [10], [12], [14], [15]. Histamine and normal saline were used as positive and negative control, respectively. The results were interpreted 15 minutes after the prick or the intradermal injection. Skin prick test was determined to be positive when wheal diameter was greater than 3 mm, and intradermal test was determined to be positive when wheal diameter increased 3 mm or more than the initial bleb [12]. The rate and factors contributing to the positivity of RCM skin test were analyzed.

### 3. Statistical Analysis

SPSS (version 19.0) was used to analyze the data. To compare the clinical features of two groups, Student t-test or Mann-Whitney test was used for continuous variables, and Chi-square test or Fisher’s exact test was used for categorical variables. To identify the risk factors related with anaphylactic shock and a positive skin test, multiple logistic regression was used. We included adjustment factors that had a P-value less than 0.1 in the univariate analysis, and other clinically important factors such as age and sex. A P-value less than 0.05 was considered statistically significant.

## Results

### 1. Clinical Characteristics of the Study Subjects and Accompanied Anaphylaxis

**Figure 1:**
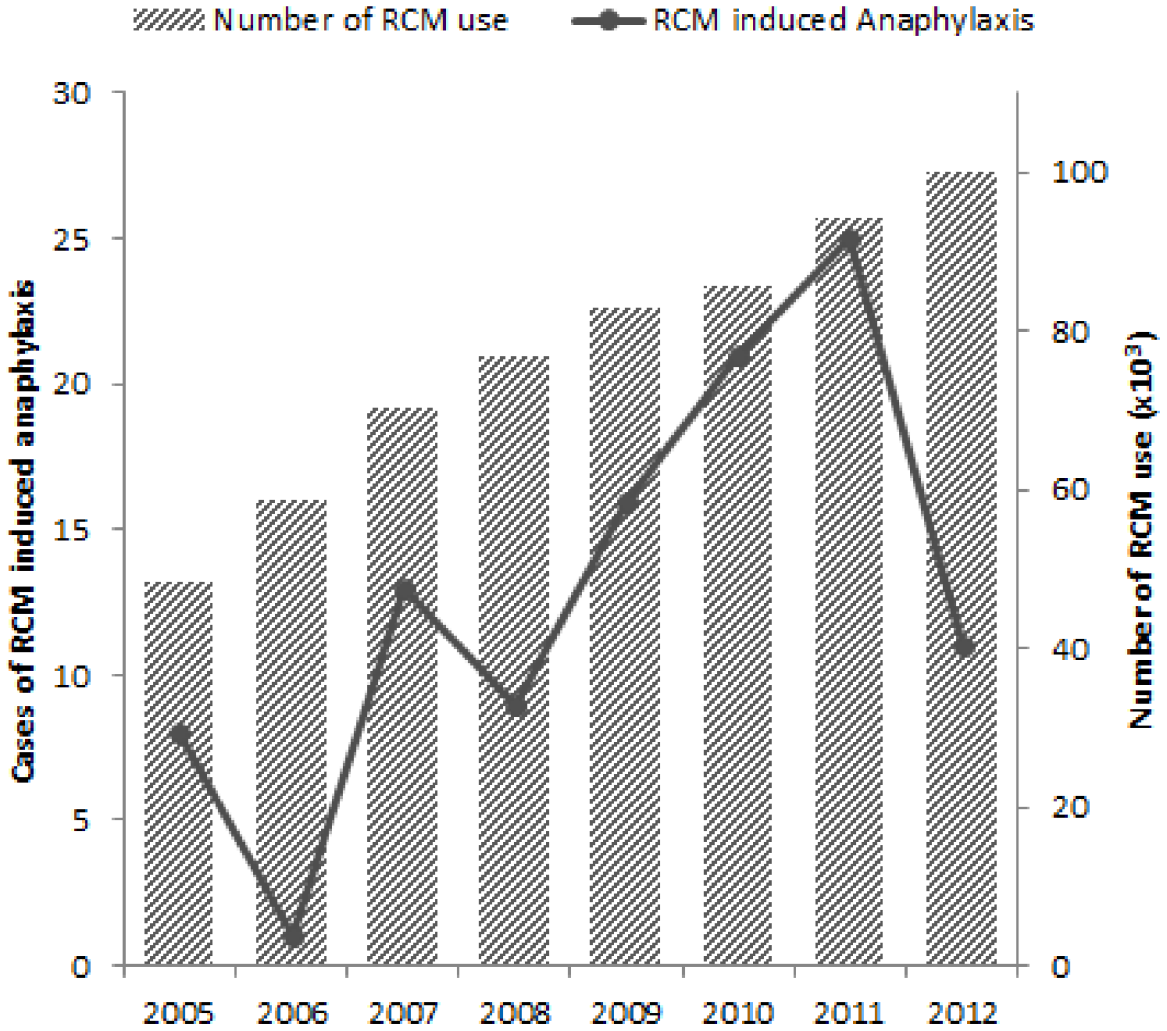
Anaphylactic reactions and total number of RCM use in every year of the study period.

A total number of contrast-enhanced CT scans during the study period was 632,513. A total of 104 cases of RCM related anaphylaxis were monitored during the study period. The incidence of contrast-induced anaphylaxis was 0.016%. As the total number of RCM use increased over the study period, the RCM related anaphylaxis also showed increasing tendency in number (Figure 1). The mean age was 55.6±13.1 years and 41.3% (43/104) of them were male (Table 1).

**Table 1:** Clinical characteristics according to the development of hypotension.

	Total (N, %)	Anaphylactic shock (N, %)	Normotensive anaphylaxis (N, %)	P-value
Number	104	78	26	
Age (years)*	55.6±13.4	57.4±13.2	50.1±13.0	0.024
Male gender, %	43 (41.3)	35 (44.9)	8 (30.8)	0.254
Number of previous exposure to RCM*	4.3±7.1	5.1±7.8	1.9±3.3	0.004
None	36 (34.6)	24 (30.8)	12 (46.2)	0.369
1	17 (16.3)	13 (16.7)	4 (15.4)	
≥2	51 (49.0)	41 (52.6)	10 (38.5)	
Previous RCM reactions	21/68 (30.9)	18/54 (33.3)	3/14 (21.4)	0.362
WBC count (/µL)	5,844.7±1,883.8	5,886±1,921	5,617±1,720	0.668
Eosinophil count (/µL)	62.0±84.4	64.2±86.9	50.0±71.4	0.574
Hypersensitivity Symptoms				
Skin symptoms	69 (66.3)	49 (62.8)	20 (76.9)	0.235
Urticaria/erythema	53 (51.0)	36 (46.2)	17 (65.4)	0.114
Angioedema	34 (32.7)	21 (26.9)	13 (50.0)	0.052
Respiratory symptoms*	50 (48.1)	32 (41.0)	18 (69.2)	0.022
Dyspnea^†^	42 (40.4)	24 (30.8)	18 (69.2)	0.001
Cardiovascular symptoms^†^	88 (84.6)	78 (100.0)	10 (38.5)	<0.001
Gastrointestinal symptoms	20 (19.2)	16 (20.5)	4 (15.4)	0.775
Underlying allergic diseases	12 (11.5)	8 (10.3)	4 (15.4)	0.464
Radiocontrast media^‡^				
Iopromide*	60 (57.7)	51 (65.4)	9 (34.6)	0.011
Iopamidol	12 (11.6)	8 (10.3)	4 (15.4)	0.726
Iomeprol	11 (10.6)	8 (10.3)	3 (11.5)	1.000
Iohexol	7 (6.7)	3 (3.8)	4 (15.4)	0.064
Iobitridol	3 (2.9)	3 (3.8)	0 (0.0)	0.571
Iodixanol	4 (3.8)	3 (3.8)	1 (3.8)	1.000
Unidentified agents*	7 (6.7)	2 (2.6)	5 (19.2)	0.010
Positive skin test^†^	33/51 (64.7)	27/33 (81.8)	6/18 (33.3)	0.001
Skin prick test	1/51 (2.0)	1/33 (3.0)	0/18 (0.0)	1.000
Intradermal test^†^	33/51 (64.7)	27/33 (81.8)	6/18 (33.3)	0.001

Continuous variables are expressed as mean ± standard deviation.

*P<0.05, ^†^P<0.01. ^‡^Among the total 104 subjects, radiocontrast media involved in anaphylaxis could not be identified in seven patients who had experienced anaphylaxis prior to the introduction of electronic medical recording system.

The median number of previous RCM exposures was 1.0 (interquartile range (IQR), 0.0–5.0) before the development of the first anaphylaxis. While anaphylaxis developed at the first exposure to RCM in 34.6% (36/104) of patients, 65.4% (68/104) of patients experienced anaphylaxis on repeated exposure to RCM and 21 of 68 (30.9%) had a milder form of hypersensitivity reactions in previous exposure to RCM.

Among hypersensitivity symptoms present in patients with anaphylaxis, cardiovascular symptoms were the most common (88/104, 84.6%), followed by skin symptoms (69/104, 66.3%) and respiratory symptoms (50/104, 48.1%) (Table 1). Most symptoms occurred within several minutes after the RCM injection. Seventy-eight patients experienced anaphylaxis with hypotension (anaphylactic shock) and 26 patients had anaphylaxis without hypotension.

### 2. Comparison of Clinical Characteristics According to the Development of Hypotension

**Figure 2:**
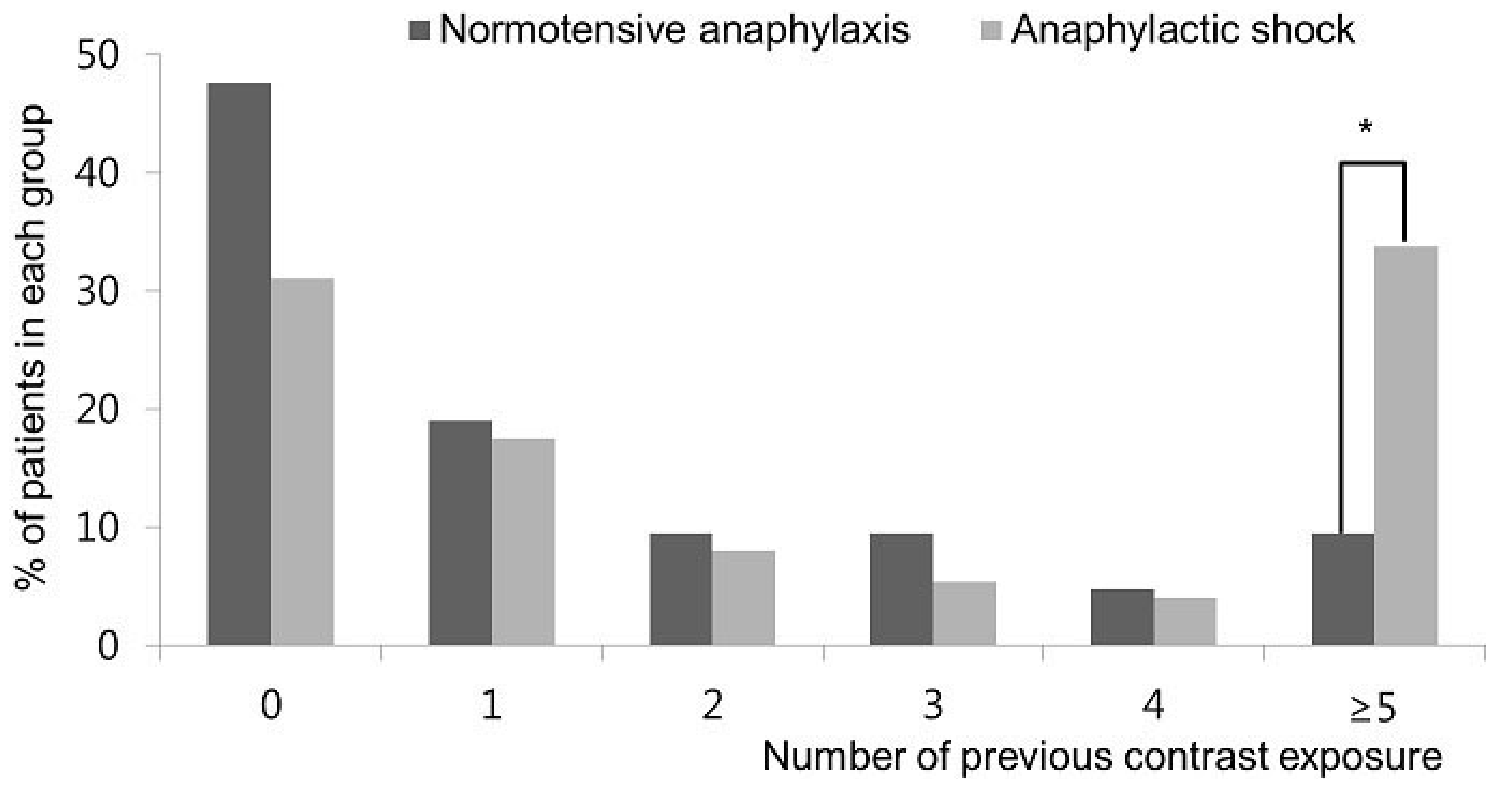
Comparison of the number of contrast exposures according to the presence of hypotension. *p<0.05.

Compared to anaphylactic patients without hypotension, patients who presented with anaphylactic shock were older (57.4 vs. 50.1 years, *p* = 0.026) and had significantly higher number of previous RCM exposures (5.1±7.8 vs. 1.9±3.3, *p* = 0.004). Of note, the number of patients who underwent previous CT more than two times was 52.6% and 38.5%, respectively in anaphylactic patients with and without hypotension. Especially, the proportion of previous exposure to RCM more than 5 times showed significant difference between anaphylactic patients with and without hypotension (35.4% vs. 9.1%, *p* = 0.018, Figure 2).

In terms of the causative contrast agent, hypotension was more frequent among anaphylaxis related to iopromide compared to other agents (85.0% vs. 61.4%, *p* = 0.011). Iopromide use was more frequently observed in patients with hypotension than in patients without it among patients with anaphylaxis (65.4% vs 34.6%, *p* = 0.011). With multiple logistic regression analysis after adjustment of age, sex, diabetes, and number of previous contrast exposure, iopromide use was still a risk factor for an anaphylactic shock (OR 3.088, 95% confidential interval (CI) = 1.078–8.843, *p* = 0.036).

### 3. Comparison of Clinical Characteristics According to Skin Test Positivity

Fifty-one patients with anaphylaxis followed the recommendation of allergists and underwent RCM skin test and the other 53 patients refused to perform skin test because they did not have a scheduled follow-up CT in the near future.

The mean interval between the time of anaphylaxis and skin test was 14.8 months (IQR 3.4–38.9). Skin test was performed in 41% of patients within one year since they experienced anaphylaxis. The remaining patients underwent skin test at the time when more than one year passed since anaphylaxis occurred (IQR 21.8–64.1).

Among those 51 patients with RCM skin test results, 33 (64.7%) had a positive response to at least one RCM while 18 patients (35.3%) did not show positivity to any RCM tested. In anaphylactic patients accompanied by hypotension, skin test showed 81.8% positivity. Among 33 patients with positive RCM skin test results, mean 1.1 contrast media (1.1±1.1) were positive per person.

Precise information on the culprit RCM was available in 29 patients. Twenty-two (75.9%) patients showed positivity to RCM including their culprit RCM; 14 patients showed single positivity to the culprit RCM; 8 patients showed positivity to other RCMs in addition to the culprit one. Another 7 patients responded to RCM agents other than the culprit one.

The positivity rate of skin test for each contrast agent is varied from 0.0% to 100.0% (Table 2). Iobitridol showed the highest sensitivity (100%) followed by iopromide (59.3%) and iodixanol (50.0%). However, all 5 patients who experienced iohexol induced anaphylaxis were negative in skin test with iohexol.

**Table 2:** Sensitivity and false negative rate on skin test.

Causative RCM	Sensitivity		False negative rate	
	To any RCM N (%)	To culprit RCM N (%)	To any RCM N (%)	To culprit RCM N (%)
Iopromide	20/27 (74.1)	16/27 (59.3)	7/27 (25.9)	11/27 (40.7)
Iopamidol	2/5 (40.0)	1/5 (20.0)	3/5 (60.0)	4/5 (80.0)
Iomeprol	4/5 (80.0)	2/5 (40.0)	1/5 (20.0)	3/5 (60.0)
Iohexol	1/5 (20.0)	0/5 (0.0)	4/5 (80.0)	5/5 (100.0)
Iobitridol	2/2 (100.0)	2/2 (100.0)	0/2 (0.0)	0/2 (0.0)
Iodixanol	1/2 (50.0)	1/2 (50.0)	1/2 (50.0)	1/2 (50.0)
Total	30/46 (65.2)*	22/46 (47.8)	16/46 (32.6)	24/46 (52.1)

*Five patients in whom causal contrast media could not be identified were excluded from this analysis among 51 patients with skin test results.

Iopromide, iopamidol, iomeprol, iohexol, and iobitridol are low-osmolar contrast media. Iodixanol is an iso-osmolar contrast media.

In patients with a positive RCM skin test, hypotension (79.4% vs. 35.3%, *p* = 0.004) and gastrointestinal symptoms (28.1% vs. 0.0%, *p* = 0.047) were more frequent compared to patients who had a negative RCM skin test. With multiple logistic regression analysis after adjustment by age, sex and diabetes, the presence of hypotension was a characteristic associated with a RCM skin test positivity (OR 10.0, 95% CI 2.105–47.098, *p* = 0.004). However, skin test positive rate was not different according to the history of previous RCM hypersensitivity reactions, accumulated number of exposures to the RCM, and underlying allergic disease.

## Discussion

Incidence of anaphylaxis is increasing rapidly and known to be 4–50/100,000 person-years [16]. In adults, drugs are the most common cause of anaphylaxis [16], [17] and radiocontrast media was the most commonly involved drug in a study of Korean tertiary care hospital [18]. Although the incidence of RCM hypersensitivity decreased as high-osmolality ionic contrasts were replaced by low-osmolality non-ionic ones, anaphylactic death still occurred regardless of ionicity [5], [6].

Traditionally, immediate hypersensitivity reactions to RCM were considered representative of non-IgE mediated ‘anaphylactoid reaction’ since it can occur on the first exposure and does not always recur on the repeated exposure [1], [10], [11], [19]. However, a previous report showed that only 30% of immediate RCM hypersensitivity developed at the first exposure to RCM [20] and our study also revealed that only 35% of RCM induced anaphylaxis occurred at the first exposures to RCM. We found that milder hypersensitivity symptoms heralded anaphylaxis in 1/3 of the patients on preceding exposure to RCM. Multiple exposures and a previous hypersensitivity reaction prior to RCM induced anaphylaxis suggest that an immunologic mechanism may have some role in the development of some RCM induced anaphylaxis.

Anaphylaxis is a severe, life-threatening systemic hypersensitivity reaction involving at least two or more organs at the same time. However, diagnosis of anaphylaxis can be made if sudden hypotension develops after exposure to a known allergen. Based upon symptoms, anaphylaxis can be classified into mild, moderate, and severe grade. [21] When hypotension occurs as a manifestation of anaphylaxis either as a sole feature or with other symptoms, physicians should pay attention to the development of potential cardiovascular collapse which is the main cause of mortality in anaphylaxis [22]. There are several known risk factors for a severe RCM hypersensitivity such as previous history of RCM hypersensitivity, asthma, allergies requiring medical treatment, use of beta-adrenergic blockers, female gender, Indian and Mediterranean ethnicity, and malignant tumor [23]. However, there was no data on the risk factors for the development of hypotension in anaphylaxis. In this study, we reported risk factors for anaphylactic shock such as older age, previous multiple exposures to RCM, iopromide use. However, we do not have a clear picture of what the overall anaphylaxis rate is using iopromide or the other study contrast agents, since patients without anaphylaxis are not included in the study. Secondly, the number of administrations of other contrast agents was too small to provide statistically significant results. In other words, we cannot conclude from this study that iopromide is more likely to cause anaphylaxis than any of the other contrast agents, but among the anaphylactic patients, iopromide was associated with more severe forms of anaphylaxis (anaphylaxis with hypotension). In addition, anaphylaxis with hypotension showed stronger association with RCM skin test positivity than anaphylaxis without hypotension. Although skin test positivity might be the result of direct mast cell activation by RCM, it is more likely that IgE mediated hypersensitivity may have a role in the development of RCM induced anaphylaxis when presented with hypotension.

Skin test is widely used to identify the causative agents in IgE mediated hypersensitivity [12], [15], [16]. Previously, sensitivity of the intradermal test was reported as high as 73% when performed with undiluted solutions [20]. However, this result may have been overestimated by irritation with undiluted RCM and a 1∶10 solution has been preferred for intradermal test with RCM in general. The positive rate of the intradermal skin test was variable and reported as low as 4.2% among patients with RCM hypersensitivity [10]. On the other hand, data from the European Network of Drug Allergy multicentre study demonstrated a 50% positive rate of RCM skin test in immediate reactors [14]. Recently, Kim et al. reported that a significantly higher sensitivity positive rate of RCM skin test in severe immediate reactions (57.1%) compared with mild (12.9%) and moderate reactions (25.0%) and suggested their modest utility in evaluating severe adverse reactions retrospectively [24]. In this study, we observed much higher positive rate of RCM skin test in patient with RCM induced anaphylaxis (64.7%). Positive rate went up as high as 81.8% among patients with anaphylactic shock and it is the highest value ever reported in RCM hypersensitivity. Three quarter of patients who showed skin test positivity responded to the very same RCM used at the time of anaphylaxis and cross reactivity rate to other RCMs was low. These findings suggest that a substantial proportion of patients with RCM induced anaphylaxis, especially anaphylactic shock, may have specificity to causative agents and skin tests can provide information on the safe substitutes. However, considering negativity in one third of patients, skin test is not helpful to choose safe alternative RCMs in some populations and clinical reasoning is needed on interpreting the results.

Although we could not perform skin test in negative controls, skin test positivity in the negative control is known to be negligible. There are several studies which elucidated very low positivity of skin test in the negative controls. Brockow et al. reported that positivity of skin test was 0.0% (0/11)–4.2% (3/71) in the negative controls [14]. Kim et al. performed RCM skin testing on 1,048 Korean subjects before contrast-enhanced CT and found only 1 case of positive immediate skin test (0.09%) [24].

There are several limitations in this study. The main limitation is its retrospective design and underreporting of adverse reaction to spontaneous reporting systems. Another limitation is the lack of information on the number of individual RCM used in contrast-enhanced CT during the study period. Thus, large scale prospective studies including sufficient number of patients reacting to each RCM are needed in order to define the exact incidence and risk factors of RCM induced anaphylaxis.

## Conclusion

RCM induced anaphylactic shock is related with multiple exposure to RCM and skin test positivity to RCM.
